# Understanding single stranded DNA gaps: from formation to fate

**DOI:** 10.1042/BCJ20253455

**Published:** 2026-03-31

**Authors:** Sonal Garg, George-Lucian Moldovan

**Affiliations:** Department of Molecular and Precision Medicine, The Pennsylvania State University College of Medicine, Hershey, PA 17033, U.S.A.

**Keywords:** Chemotherapy, DNA damage response, DNA replication and recombination, DNA synthesis and repair, Drug therapy

## Abstract

Single-stranded DNA gaps (ssDNA gaps) have emerged as a potential indicator of therapeutic response in cancer. Accumulation of ssDNA gaps is associated with increased sensitivity of cancer cells to genotoxic therapies like PARP inhibitors (PARPi) and cisplatin chemotherapy. However, efficient repair or suppression of ssDNA gap formation is associated with therapy resistance and treatment failure. Therefore, understanding how ssDNA gaps form and are repaired can help identify biomarkers that can guide new treatment strategies to overcome resistance. In this review, we discuss different sources of ssDNA gap formation and the repair mechanisms that have been characterized to date. We bring together current knowledge on how these gaps are processed and what their ultimate fate may be. Finally, we discuss how established drugs like PARPi, hydroxyurea, and platinum compounds, induce and/or exploit ssDNA gaps. Throughout this review, we highlight ssDNA gaps as a potential therapeutic vulnerability that can be used to advance personalized cancer therapy.

## Introduction

DNA replication is tightly regulated to ensure accurate duplication of the genome. However, DNA replication is constantly challenged by obstacles that can block replicative DNA polymerases. These obstacles can arise from intrinsic sources like DNA–protein complexes, secondary DNA structures, or replication-transcription conflicts, as well as from extrinsic sources like UV radiation, chemical agents, and chemotherapeutic drugs [1–4]. The stalling of the DNA polymerase due to these obstacles is referred to as replication stress. It can generate a variety of toxic DNA lesions like bulky adducts, thymidine dimers, interstrand cross-links, and abasic sites that block replication fork progression, resulting in replication fork stalling.

**Figure 1:**
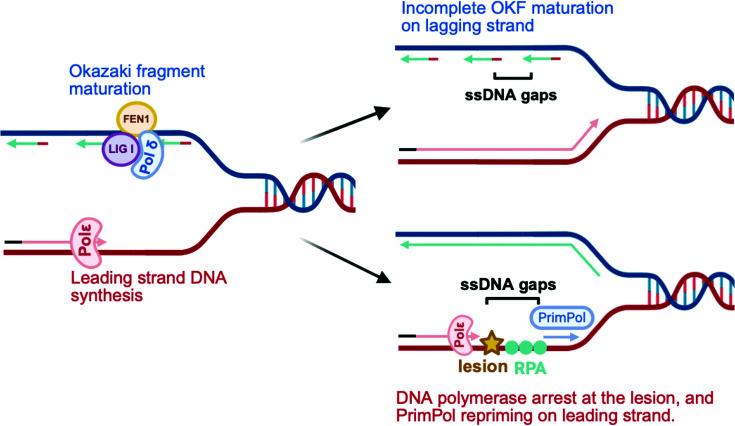
Formation of ssDNA gaps on leading and lagging strands during DNA replication On the lagging strand, incomplete processing of Okazaki fragments (OFs) results in gaps between adjacent OFs. On the leading strand, polymerase stalling at DNA lesions triggers repriming by PrimPol, leaving a gap behind the lesion. Created in BioRender. Moldovan, G. (2026) https://BioRender.com/a0xlqxm.

To preserve the genomic integrity and ensure cellular survival, cells employ a network of signaling and repair pathways known as DNA damage response (DDR). DDR signals the cells to activate the cell cycle checkpoints and recruit DNA damage repair pathways such as base excision repair (BER) and nucleotide excision repair (NER) that act directly to fix the damage by excising the lesion [5–7]. However, when cells encounter a lesion in S-phase, replicative DNA polymerases are unable to synthesize past the damage, leading to replication stress that, if left unresolved, can result in replication fork collapse and cell death. To avoid prolonged fork stalling, DNA damage tolerance (DDT) pathways are activated that allow replication to continue despite the damage. Key DDT pathways include fork reversal, “on the fly” translesion DNA synthesis (TLS), and fork repriming. Fork reversal temporarily remodels the fork by the activity of fork remodelers like SMARCAL1 and HLTF [8]. “On the fly” TLS uses specialized polymerases to bypass the lesion and continue replication [9]. Fork repriming involves a specialized primase polymerase, PrimPol, that reprimes downstream of the lesion, allowing replication to continue while leaving behind single-stranded DNA gaps (ssDNA gaps) [10] (Figure 1). In healthy cells, these pathways coordinate DNA replication and DNA repair to limit the accumulation of genomic errors. However, cells carrying mutations in DNA repair or tolerance pathways fail to efficiently resolve DNA damage, which can increase the likelihood of accumulating mutations that range from small-scale point mutations to structural rearrangements and other chromosomal changes.

These alterations in DNA repair genes drive genome instability, a hallmark of cancer, defined by the tendency of cancer cells to acquire genetic mutations that enable them to grow uncontrollably [11,12]. Mutations in DNA repair genes can promote cancer either as inherited (germline) or acquired (somatic) events. Inherited mutations can predispose individuals to cancer syndromes, such as hereditary breast and ovarian cancer caused by BRCA1 or BRCA2 mutations, while somatic mutations that accumulate during lifetime can contribute to sporadic cancers.

Genome instability is therefore a common feature among cancer cells and represents an important therapeutic vulnerability. Genotoxic chemotherapies, including platinum-based therapy, topoisomerase inhibitors, and alkylating agents, exploit this weakness of cancer cells by inducing additional DNA damage and promoting cell death. Early paradigms in the field were shaped by studies involving ionizing radiation that induces double-stranded DNA breaks (DSBs). These studies established a strong link between DSB accumulation and therapy response [13,14]. Therefore, DSBs were considered the dominant cytotoxic lesions, as they can lead to large-scale chromosomal rearrangements and promote cell death. BRCA deficiency can predict therapy response due to the role of BRCA proteins in homologous recombination (HR), the high-fidelity pathway for DSB repair. BRCA-deficient cells were found to be highly sensitive to agents like cisplatin and PARP inhibitors (PARPi) [15–17]. To explain this, it was proposed that these therapeutic agents generate primary lesions or repair intermediates that are converted into DSBs during DNA replication. Cisplatin induces DNA inter- and intra-strand cross-links that can block replication and ultimately generate DSBs, while PARPi’s were thought to act primarily by trapping PARP on DNA, creating lesions that stall replication, leading to fork collapse and DSB formation [16,18]. In addition to DSB repair deficiency, a “broken fork” model emerged, proposing that chemotherapeutic agents induce replication fork stalling that leads to fork collapse. This model included a second function of BRCA proteins in fork protection (FP), through which BRCA stabilizes stalled replication forks by preventing nucleolytic degradation [19–21]. Loss of BRCA function was therefore proposed to result in accumulation of DSBs, replication fork collapse, and ultimately cell death. Together, these observations supported that DSBs are the dominant cytotoxic lesions driving genome instability and therapeutic response in BRCA-mutated tumors.

However, later studies challenged this model and suggested that HR and/or FP may not be sufficient to explain therapy response. Restoring FP through MRE11 inhibition or SMARCAL1 depletion fails to confer cisplatin resistance, suggesting that replication fork collapse is not the major consequence of treatment [18]. Moreover, it was reported that HR-proficient cells with mutant RAD51 remain sensitive to cisplatin, indicating that HR alone is not always sufficient for chemoresistance [22]. BRCA-deficient cells were also highly sensitive to agents such as hydroxyurea and oxaliplatin (another platinum drug), which do not induce DSBs [18,23]. These observations raised the possibility that DSBs might not be the primary toxic lesion driving chemosensitivity.

Instead, emerging evidence suggests that ssDNA gaps induced by genotoxic agents may be the main contributor to chemosensitivity in BRCA-mutated cancers [18,23,24]. Experiments involving electron microscopy and chromosome spread analyses showed that BRCA-deficient cells accumulate ssDNA gaps behind replication forks, independent of DSB formation [19,25]. These ssDNA gaps are found to arise from defects in replication gap suppression (RGS), Okazaki fragment (OF) processing, and lagging strand synthesis, highlighting an additional role for BRCA in maintaining replication integrity [22,25,26]. Importantly, ssDNA gaps are observed in response to various genotoxic agents, including cisplatin and hydroxyurea. Suppression of these gaps through restoration of RGS pathways [18,24], fork restraint [26], post-replicative TLS [27,28], or replication protein A (RPA) protection has been seen to restore therapy resistance. Several studies noticed that DSBs are formed later on as a consequence of apoptosis [18,24], suggesting that gaps may act as an early driver of chemosensitivity.

These findings suggest that ssDNA gaps may serve as a more accurate indicator of treatment response. The accumulation of ssDNA gaps makes cancer cells sensitive to chemotherapeutic agents, whereas the repair of these gaps makes them resistant. Therefore, it becomes important to understand the mechanisms responsible for ssDNA gap repair as targeting these pathways could enhance response to genotoxic drugs. This review summarizes different sources of ssDNA gap formation, as well as the pathways responsible for repairing these gaps. We examine the consequences of unfilled gaps on genome stability and cell survival, and highlight the genotoxic agents associated with ssDNA gap induction.

## Sources of ssDNA gaps

### Lagging strand ssDNA gaps

During DNA replication, the lagging strand is synthesized discontinuously as short OFs. First, DNA polymerase alpha primase (Pol α) synthesizes short RNA primers on the lagging strand and facilitates initial extension. Since Pol α is a low-fidelity polymerase and lacks the proofreading 3′–5′ exonuclease activity, the extension is taken over by the high-fidelity polymerase delta (Pol δ), which generates short stretches of DNA nucleotides (150–200 bp) known as OFs. As a consequence of discontinuous synthesis of the lagging strand, nicks in the phosphosugar backbone are generated between adjacent fragments. In healthy cells, these nicks are efficiently sealed during OF processing for complete strand maturation [29–31]. In normal OF processing, Flap endonuclease 1 (FEN1) and RNase H2 remove RNA primers, Pol δ fills the resulting gaps, and DNA ligase 1 (LIG1) seals the nicks, generating a continuous lagging strand. Disruption of this coordinated OF processing, due to mutations or functional defects in FEN1, LIG1, or Pol δ, leads to incomplete maturation of the lagging strand and the accumulation of ssDNA gaps [29] (Figure 1). In addition to defective OF processing, ssDNA gaps can arise when Pol δ encounters DNA lesions during lagging-strand synthesis. In such cases, Pol δ can skip the lesion and continue DNA synthesis at the next OF using its RNA primer, leaving behind ssDNA gap opposite of the lesion [32]. Therefore, lagging-strand ssDNA gaps can originate from both impaired OF maturation and lesion bypass during replication (Figure 1).

When canonical OF processing fails, lagging-strand ssDNA gaps are repaired by a PARP1-dependent backup pathway [33]. Poly (ADP-ribose) polymerase 1 (PARP1) is a DNA repair protein that detects single-strand DNA regions. Once bound to these regions, PARP1 undergoes auto-PARylation (poly-ADP-ribosylation), generating PAR chains that serve as a recruitment signal for XRCC1 (X-ray repair cross-complementing protein 1). XRCC1 is a scaffold protein that provides a platform for DNA polymerase β (Pol β), which fills in the gap, followed by DNA ligase 3 (LIG3), which ligates the resulting nicks [33,34]. This PARP1–XRCC1–LIG3 axis therefore compensates for defects in canonical OF processing.

The PARP1–XRCC1–LIG3 axis is also responsible for repairing gaps that form during BER [35,36]. During BER, the damaged base is excised, and the sugar-phosphate backbone is incised by AP endonuclease 1 (APE1), generating an ssDNA region. PARP1 recognizes these BER intermediates and recruits XRCC1 and LIG1. Therefore, PARP1 plays a central role in maintaining lagging-strand integrity and efficient BER. Inhibition of PARP1 by PARPi can prevent the repair of both OF gaps and BER-associated ssDNA gaps, leaving them unrepaired and sensitizing cells to replication stress and genomic instability.

Efficient completion of PARP1-mediated repair requires timely removal of PARP1 from DNA. Poly (ADP-ribose) glycohydrolase (PARG) removes PAR chains from PARP1, known as dePARylation, allowing PARP1 and repair proteins to dissociate and resume replication [37]. Loss of PARG1 prevents dePARylation, and therefore, PARP1-associated DNA repair proteins remain trapped on the DNA. This leads to the formation of protein–DNA complexes that act as a physical block to the replication machinery, further contributing to replication stress [38]. Consistent with this, PARG inhibitors have been implicated in the treatment of BRCA-mutant tumors. Studies suggest that the central cause of PARGi sensitivity in cancer cells is the accumulation of ssDNA gaps [39,40].

Recent studies have identified additional factors that regulate PARP1-dependent gap repair. Among them, TIMELESS (TIM), a core component of the replication FP complex, has been shown to coordinate PARP1-mediated repair. TIM interacts with PARP1 at replication forks and promotes PARylation, allowing the recruitment of XRCC1 and enhancing the repair of ssDNA gaps [41]. In addition, polynucleotide kinase phosphatase (PNKP) has been implicated to play a role in filling lagging strand ssDNA gaps. PNKP has both 3′ DNA phosphatase and 5′ DNA kinase activity. At a DNA nick with 3′-phosphate (3′-P) or 5′-hydroxyl (5′-OH) ends, the DNA ends are not chemically compatible for ligation. PNKP, through its end processing activity, converts these ends into 5′-P and 3′-OH, and makes them compatible for ligation by DNA ligases [42]. This role of PNKP has been established in BER, non-homologous end joining, and single-stranded break (SSB) repair. The recruitment of PNKP to the ssDNA gaps between OFs is mediated by CDK-dependent phosphorylation, highlighting a cell-cycle-controlled mechanism [43].

### Leading strand ssDNA gaps

In eukaryotic cells, the leading strand is synthesized by the high-fidelity replicative DNA polymerase epsilon (Polε) [44–46]. Polε has a small active site that cannot accommodate damaged bases. Therefore, when Polε encounters DNA lesions or replication obstacles on the template strand, it stalls the replication fork. Under these circumstances, the CMG helicase can continue unwinding the DNA, creating ssDNA regions [47,48]. These regions are rapidly coated by RPA, which stabilizes ssDNA and protects it from nucleolytic degradation. To overcome stalled replication, cells employ PrimPol, a specialized primase-polymerase. PrimPol is directed to the stalled forks through its interaction with RPA, where it synthesizes a short RNA primer downstream of the lesion and allows replication to continue. However, PrimPol has limited processivity and can incorporate only a few nucleotides before Pol ε takes over to resume high-fidelity DNA synthesis [50–52]. Although PrimPol repriming allows replication to continue, it leaves behind ssDNA gaps opposite the lesion, which persist behind the replication fork [52,53] (Figure 1). PrimPol-mediated repriming is thought to mostly occur during leading strand DNA synthesis, since lagging strand synthesis is inherently discontinuous.

PrimPol repriming is triggered in response to several types of replication stressors, including bulky DNA adducts such as UV-C [54] and BPDE-induced lesions [55], and DNA cross-links caused by cisplatin or mitomycin C [56,57]. Additionally, small base modifications like uracil lesions generated by APOBEC3A or oxidized bases that are processed by UNG and SMUG1 glycosylases also promote PrimPol-dependent ssDNA gap formation [58,59]. PrimPol activity becomes particularly important when other DDT mechanisms, such as fork reversal and “on the fly” TLS, are compromised. For instance, loss of fork remodeling factors like SMARCAL1, HLTF, ZRANB3, or the chromatin regulator CARM1 up-regulates PrimPol as a compensatory mechanism [53,57]. In addition, the ATR–Chk1 signaling phosphorylates PrimPol at serine 255 during replication stress, promoting its recruitment to the nascent DNA [60]. Recent studies have also indicated the role of chromatin regulators, showing that histone chaperones CAF-1 and ASF1 promote ssDNA gap accumulation by facilitating PrimPol loading onto nascent DNA [61]. On the other hand, several factors can down-regulate PrimPol activity to prevent excessive gap formation. For example, BRCA2 blocks the recruitment of PrimPol by interacting with MCM10 [62], while the CST complex (CTC1–STN1–TEN1) limits PrimPol loading after UV damage [63]. Additionally, p53 (tumor suppressor that regulates cell division) and DNA Polymerase iota (Polι) form a complex that suppresses PrimPol activity [64].

## Pathways involved in filling ssDNA gaps

**Figure 2:**
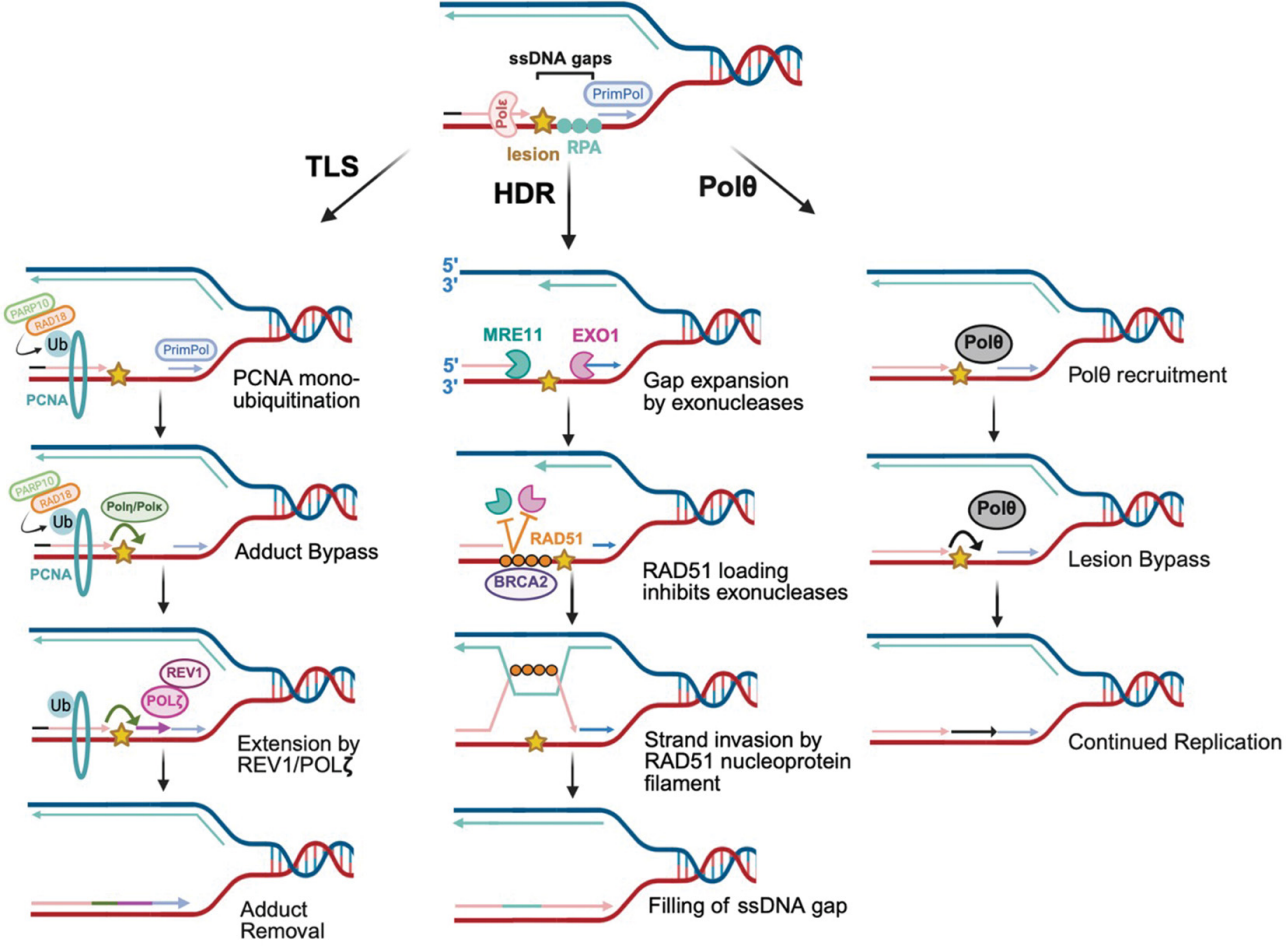
Different pathways employed by cells to repair ssDNA gaps TLS uses specialized polymerases that bypass the DNA lesion and subsequently fill post-replicative ssDNA gaps. HDR uses the sister chromatid as a template to accurately fill ssDNA gaps. POLθ can also bypass DNA lesions to fill ssDNA gaps. For simplicity, these pathways are illustrated on the leading strand, but they can also occur on the lagging strand. Created in BioRender. Moldovan, G. (2026) https://BioRender.com/oeceup8.

Post-replicative ssDNA gaps are formed during DNA replication and must be repaired to maintain genome stability. Cells employ different mechanisms to fill these gaps, among which TLS and homology-directed repair (HDR) are the two well-established post-replicative gap-filling mechanisms. In addition to these, there is evidence suggesting a separate, DNA polymerase theta (Polθ)-mediated gap-filling process (Figure 2).

### TLS mediated repair of ssDNA gaps

TLS plays two important roles in maintaining genome integrity. One is “on-the-fly” TLS, which occurs co-replicatively, where TLS polymerases bypass DNA lesions directly at the stalled replication fork [27]. The second is post-replicative gap filling, where TLS fills in ssDNA gaps that are left behind due to PrimPol repriming or impaired OF processing [28]. These post-replicative gaps are coated by RPA, which stabilizes the ssDNA region and recruits the downstream repair factors. The E3 ubiquitin ligase RAD18 directly binds to RPA-coated ssDNA [28] and monoubiquitinates PCNA (proliferating cell nuclear antigen), a sliding clamp that encircles the DNA and provides a platform for DNA polymerases. Our laboratory has shown that PARP10, an ADP-ribosyl transferase, interacts with RAD18 and promotes its recruitment to the ssDNA gap [65]. Monoubiquitinated PCNA acts as a molecular signal for the recruitment of low-fidelity TLS polymerases, polymerase kappa (Polκ), polymerase eta (Polη), polymerase iota (Polι), polymerase zeta (Polζ), and polymerase REV1. These polymerases bind to mono-ubiquitinated PCNA via their ubiquitin-binding motifs and initiate gap filling. Post-replicative TLS is known to proceed in two steps: first, bypass TLS polymerases (Polκ, η, and ι) insert a nucleotide opposite the lesion. Then, extension TLS polymerase POLζ (composed of REV3 and REV7) continues synthesis beyond the lesion and fills the gap (Figure 2). The switch between bypass and extension polymerases is coordinated by REV1, a scaffold protein that interacts with both bypass and extension polymerases [27,58,66,67]. Disruption of the REV1–Polζ interaction impairs post-replicative TLS-mediated gap filling [68]. Unlike replicative polymerases, TLS polymerases have a larger active site that is capable of accommodating bulky DNA lesions, but lack a proofreading domain, which makes them error-prone and potentially mutagenic [32,69,70]. Moreover, different TLS polymerases bypass specific types of lesions due to differences in the shape and size of their active site. For example, Polη is capable of accommodating UV-induced dimers and cisplatin adducts, while Polκ mainly bypasses BPDE adducts [27,71,72].

Given its mutagenic potential, TLS is tightly regulated. The deubiquitinase USP1 negatively regulates TLS by removing ubiquitin from PCNA, thereby limiting TLS polymerase recruitment [73–75]. We have shown that USP1 promotes ssDNA gap accumulation by down-regulating TLS [76]. When USP1 is active, TLS is down-regulated, which promotes the accumulation of post-replicative ssDNA gaps. These ssDNA gaps require RPA binding for protection, but excessive ssDNA can deplete cellular RPA pools, leaving regions of ssDNA vulnerable to nucleolytic degradation, thereby promoting genome instability. On the other hand, inhibition of USP1 increases PCNA ubiquitination and up-regulates TLS, thus promoting ssDNA gap suppression [76].

### HDR of ssDNA gaps

While TLS is fast, it is often mutagenic due to the low fidelity of TLS polymerases. Another way used by cells to fill post-replicative ssDNA gaps is an HDR mechanism that uses the sister chromatid as a homologous donor to accurately restore genetic information [77]. Since this mechanism relies on sister chromatid, it is restricted to late S and G2 phases of cell cycle.

RAD51 is the key recombinase essential for HDR of ssDNA gaps that arise during DNA replication. These gaps are formed behind the replication fork. RAD51 is recruited to the ssDNA gaps by BRCA2. RAD51 recruitment is also facilitated by nucleolytic processing of ssDNA gaps via MRE11, EXO1, and DNA2 exonucleases, which generate ssDNA regions that serve as a substrate for RAD51 [57]. RAD51 then forms a nucleoprotein filament that searches for a homologous sequence on the sister chromatid (Figure 2). Once the sequence is identified, RAD51 mediates strand invasion of the stalled nascent strand into the newly synthesized DNA of the sister chromatid, allowing DNA polymerases to copy the correct sequence and fill the gap.

In contrast, RAD51 is also central for the repair of DSBs and collapsed replication forks via HR. HR uses the intact duplex DNA of the sister chromatid as the donor template [77]. This pathway requires extensive DNA end resection, initiated by the MRN complex (MRE11, RAD50, and NBS1) [53,78]. MRE11 recruitment to the replication fork is supported by PARP1 or the BRCA2–PTIP axis to facilitate resection of the 5′ end [79,80], while EXO1 and DNA2 resect at the 3′ end to generate 3′ ssDNA overhangs [81]. In addition, BRCA1 interacts with C-terminal binding protein (CtBP)-interacting protein (CtIP) and the MRN complex to facilitate efficient end resection [82]. After DNA end resection, the exposed ssDNA is coated by RPA, which stabilizes the strand and prevents secondary structures. BRCA2 then replaces RPA with RAD51. RAD51 filaments then perform strand invasion into the double-stranded DNA on the homologous chromosome, creating a D-loop to complete repair [77]. Thus, although RAD51 is central to both HDR of ssDNA gaps and HR of DSBs, these pathways may be distinct in terms of the structure involved.

Several factors regulate HDR-mediated ssDNA gap repair. Using Förster resonance energy transfer, RAD51 loading was shown to eliminate PCNA at the stalled replication fork, acting as a switch away from post-replicative TLS and promoting HDR-mediated gap repair [83]. The BCDX2 complex (RAD51B, RAD51C, RAD51D, and XRCC2) facilitates the formation and stabilization of RAD51 nucleofilaments on ssDNA regions [84]. RAD51 paralogs RAD55 and RAD57 also help stabilize RAD51 filaments on the ssDNA region, and thus, promote HDR-mediated gap repair [85]. We have identified HELQ and RAD52 as the regulators of HDR repair. The helicase HELQ promotes RAD51 recruitment and filament formation; however, RAD52, an ssDNA annealing factor, can anneal the invaded strand to the parental strand and prevent RAD51-mediated strand invasion. Interestingly, in cells lacking BRCA2, RAD52 can partially compensate and promote RAD51-mediated strand invasion, acting as a backup mechanism for HDR [86]. ATR signaling has also been shown to play a role in regulating HDR-mediated gap repair. After nucleolytic degradation of the DNA ends, the ssDNA is coated by RPA, which acts as a signal for ATR. Once activated, ATR prevents further activity of EXO1 and DNA2 nucleases to limit the expansion of the gaps [87,88].

Beyond its role in HDR-mediated gap repair and HR repair of DSBs, RAD51 also maintains genomic stability in other ways. It binds to abasic sites created during BER and protects them from excessive degradation by MRE11 and APE1. In the absence of RAD51-mediated protection, abasic sites can lead to persistent ssDNA gaps and replication fork collapse. Therefore, RAD51 prevents further damage by protecting abasic sites [89]. Additionally, RAD51 has been implicated in controlling DNA re-replication. When the cells experience replication stress, the replication origins can be re-fired, leading to unscheduled DNA synthesis. RAD51 bound at the replication fork slows fork progression and often induces fork reversal. This triggers MRE11-mediated degradation of re-replicated DNA, preventing overduplication [90].

### DNA polymerase theta-mediated repair of ssDNA gaps

DNA polymerase theta (Polθ) is best known for its role in theta-mediated end-joining (TMEJ), also known as microhomology-mediated end joining, where it repairs DSBs by joining resected DNA ends using short regions of microhomology [91,92]. BRCA-deficient cells rely on Polθ for DSB repair, as evidenced by up-regulated expression of Polθ in BRCA-deficient cells. Depletion of Polθ results in synthetic lethality in BRCA-deficient cells, explained by defective HR and TMEJ [93,94].

Interestingly, there is growing evidence suggesting a role of Polθ in post-replicative gap filling. Studies have shown that Polθ plays a role in suppressing ssDNA gaps, particularly those arising from incomplete processing of OFs on the lagging strand [95,96]. These gaps can result due to dysregulated Polα/primase activity or PARP inhibition. Polθ can fill these gaps using polymerase activity to extend OFs and its helicase activity to remove DNA–protein lesions that block replication [95]. Recruitment of Polθ to ssDNA gaps is mediated by PARP1 [97] or through PCNA ubiquitination [98]. By filling the ssDNA gaps, Polθ stabilizes replication forks and prevents fork reversal [95]. In the absence of Polθ, ssDNA gaps become vulnerable to the nucleolytic attack by the MRE11–NBS1–CtIP complex, leading to cytotoxic double-strand breaks [96]. Additionally, studies suggest that Polθ can bypass DNA lesions on both the leading and lagging strands through a role in TLS, though this role needs future investigation [99] (Figure 2). Given its dual function in DSB repair and ssDNA gap filling, targeting Polθ can compromise both pathways, explaining the reason behind synthetic lethality of BRCA-deficient cancer cells with Polθ inhibitors.

## What happens when ssDNA gaps are not filled?

When replication forks encounter DNA lesions, they can be reversed by DNA translocases, forming a four-way junction that allows DNA synthesis to continue on the reversed arm. When cells use fork reversal to tolerate replication stress, BRCA proteins can stabilize RAD51 filaments on the reversed arms of the replication fork and prevent nucleolytic degradation by MRE11 and EXO1 exonucleases [19,100,101]. However, in BRCA-deficient cells, the fork is not protected and can undergo nucleolytic degradation [19,102]. This protective role of BRCA proteins was considered the main reason for the chemosensitivity of BRCA-deficient cells to genotoxic agents. However, with the newer evidence that BRCA-deficient cells accumulate ssDNA gaps and that BRCA proteins play a role in filling post-replicative ssDNA gaps, it is important to understand the role of nucleases in ssDNA gap repair. Interestingly, studies show that inhibition of MRE11 suppresses ssDNA gap accumulation in BRCA-deficient cells [53,78]. This suggests that if the gaps are not filled, they are processed by exonucleases. Therefore, with loss of BRCA proteins, unfilled ssDNA gaps become a substrate for nucleases.

MRE11 is a part of the MRN complex, which plays an important role in DNA end resection during HR for DSB repair. MRE11 initiates short-range resection by first making a cut on the 5′ strand using its endonuclease activity and then trimming back toward the break in the 3′ to 5′ direction through its exonuclease activity. This initial resection is then extended through long-range resection by nucleases EXO1 or DNA2, which digest DNA in the 5′ to 3′ direction [78]. Resection by MRE11 and EXO1 generates 3′ ssDNA overhangs that are coated by RPA, allowing BRCA2 and RAD51 to mediate strand invasion and HR repair. While the roles of MRE11 and EXO1 in HR are well characterized, their role in processing ssDNA gaps is still being investigated. Evidence suggests that MRE11 resects ssDNA gaps in the 3′ to 5′ direction. On the other hand, long-range exonucleases, EXO1 and DNA2 resect ssDNA gaps in the 5′ to 3′ direction. Therefore, if the ssDNA gaps are not filled by post-replicative TLS or HDR, they can undergo bidirectional expansion by exonucleases [103,104]. Extension of ssDNA gaps can create long stretches of fragile ssDNA that contribute to genomic instability. However, whether nuclease resection of gaps follows the same short- and long-range resection as at DNA ends, and whether all gaps are subject to such nucleolytic expansion, remains unclear.

**Figure 3:**
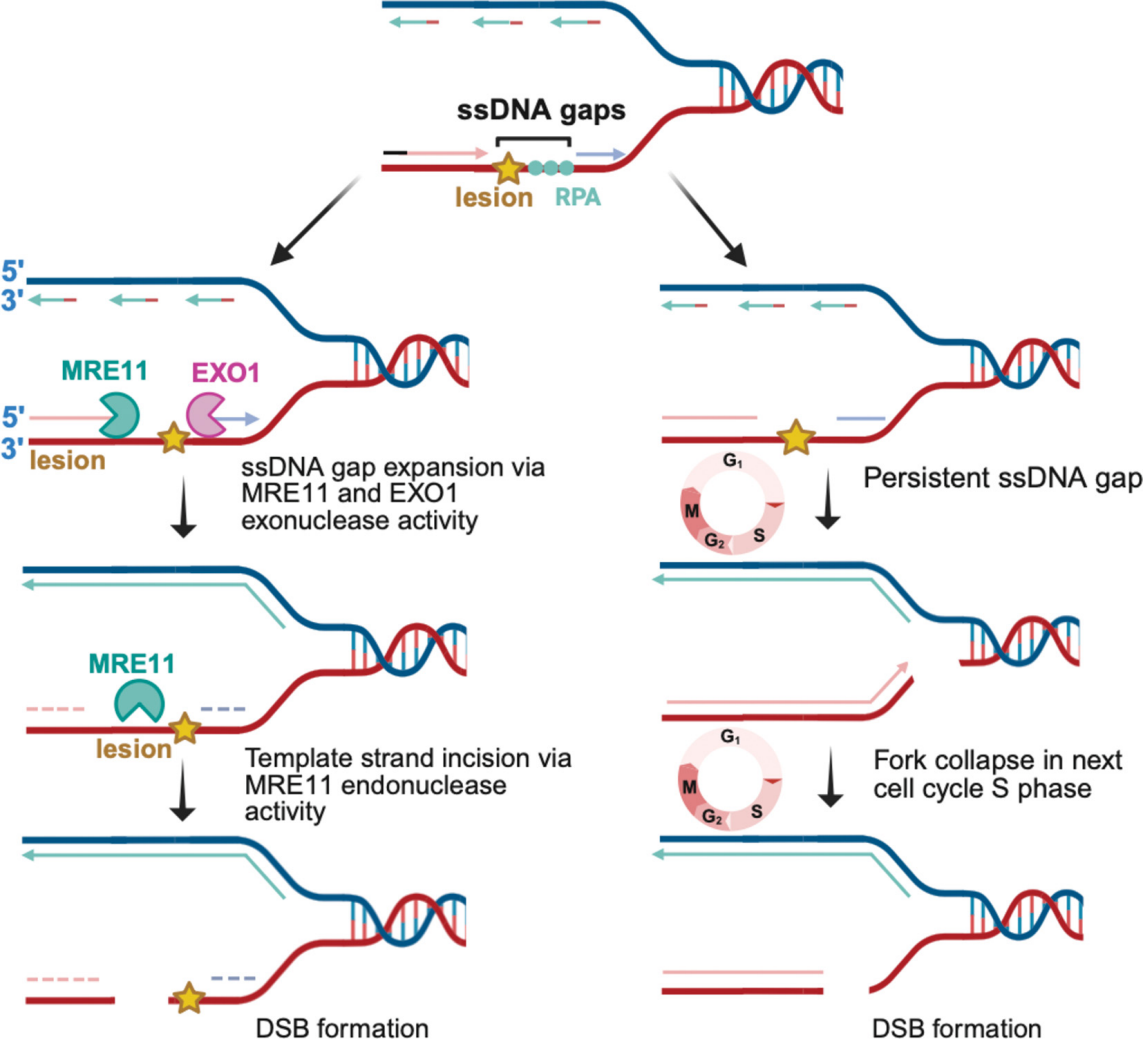
Hypothetical models for the conversion of unfilled ssDNA gaps into cytotoxic double-strand breaks The schematic shows two possibilities: direct cleavage of the template strand by MRE11, generating a DSB (left), and persistence of gaps into the next cell cycle, where replication fork collision causes fork collapse and DSB formation (right). Created in BioRender. Moldovan, G. (2026) https://BioRender.com/yliehpu.

In addition to gap expansion, another possible fate of unfilled ssDNA gaps is their conversion into DSBs [17], a highly toxic form of DNA damage that can lead to chromosomal rearrangements or cell death. The exact mechanism by which ssDNA gaps are converted into DSBs is under investigation. One model suggests that excessive resection of ssDNA gaps by exonucleases leads to the formation of long single-stranded regions that deplete nuclear RPA, leaving unprotected ssDNA that is vulnerable to further damage [105]. In this context, it is hypothesized that MRE11, through its endonucleolytic activity, cleaves the intact template strand opposite the gap and converts the single-stranded lesion into a DSB [103,106] (Figure 3). On the other hand, SOME studies propose that these nucleases work differently on ssDNA gaps than on the DSBs. While MRE11, EXO1, and DNA2 exonucleases promote bidirectional gap expansion (similarly as at DNA ends), it is possible that MRE11 may not nick the template strand [104,107]. As a result, these ssDNA gaps persist during the current cell cycle and are converted into DSB during the next round of replication, when an incoming fork collides with the gap and collapses (Figure 3). In both scenarios, BRCA-proficient cells prevent DSB formation through FP and efficient gap filling, whereas BRCA-deficient cells accumulate unrepaired gaps that are prone to DSBs. Therefore, both hypotheses highlight the importance of efficient gap filling, as a failure to do so can not only result in gap expansion but can also lead to more severe forms of DNA damage. It is possible that both mechanisms occur in cells, some gaps may be directly cleaved by MRE11, while others are converted to DSBs during replication. However, how cells decide between these two outcomes remains to be understood. Nevertheless, studies have shown that ssDNA gaps by themselves may not be cytotoxic, but rather it is their processing into DSBs that results in cellular sensitivity to ssDNA gap-inducing agents. This is perhaps in line with recent work showing that BRCA2 mutants defective in RAD51 filament stabilization accumulate ssDNA gaps and exhibit FP defects but retain DSB repair and do not display increased sensitivity to drugs like cisplatin and PARPi [108]. These findings argue that the ability of the BRCA pathway to perform DSB repair, rather than FP and gap suppression, correlates with drug sensitivity.

Although excessive resection by exonucleases can lead to DSBs, controlled resection is required for initiating HR. Therefore, it becomes important to regulate the activity of nucleases at ssDNA gaps. MRNIP (MRN-interacting protein) has been identified as a negative regulator of MRE11. It was initially shown to protect reversed replication forks from MRE11-mediated degradation. However, more recent studies suggest that MRNIP also prevents the formation of post-replicative ssDNA gaps, and loss of MRNIP results in the accumulation of ssDNA gaps [109]. Therefore, in the absence of MRNIP, unregulated MRE11 activity can expand ssDNA gaps and also increase the probability that these gaps persist and convert into DSBs.

## Chemotherapeutic drugs reported to induce ssDNA gaps

Accumulation of ssDNA gaps contributes to genome instability and can sensitize tumor cells to a variety of genotoxic therapies. PARPi are an important example. Clinically approved for the treatment of BRCA-deficient tumors, PARPi promote cell death through several mechanisms [15,16,110]. They disrupt the PARP1-mediated SSB repair pathway and lead to the accumulation of SSBs that are eventually converted into DSBs. Second, PARPi bind to the catalytic site of PARP1 and prevent its auto-PARylation. In the absence of auto-PARylation, PARP1 cannot dissociate from DNA and remains bound at sites of damage. This creates a bulky DNA lesion that blocks replication fork progression and induces fork stalling [110–112]. BRCA-proficient cells can rescue the stalled fork and repair resulting DSBs through their role in FP and HR [113], respectively. However, BRCA-deficient cells lacking both HR and FP are sensitive to PARPi. Recent studies highlight that the sensitivity of BRCA-deficient tumors to PARPi can also be attributed to the role of BRCA in ssDNA gap repair. The accumulation of ssDNA gaps is now recognized as a primary source of PARPi sensitivity in BRCA-deficient cells. For example, dysfunction of POLA1, a subunit of DNA polymerase α-primase that regulates fork speed, can accelerate forks and promote ssDNA gap formation under PARPi treatment [114].

Platinum-based chemotherapies like cisplatin and carboplatin also induce ssDNA gaps. Cisplatin chemotherapy is widely used to treat several cancers, including ovarian, bladder, and head-and-neck tumors. Approximately 95% of cisplatin-induced DNA damage consists of intrastrand adducts, where two purine bases on the same DNA strand are covalently linked [115]. These lesions lead to the arrest of high-fidelity replicative DNA polymerases, resulting in replication fork stalling. To bypass these lesions, cells employ PrimPol, which reinitiates DNA synthesis downstream of the adduct and leaves behind ssDNA gaps. Therefore, processing of cisplatin-induced lesions generates ssDNA gaps. Additionally, replication inhibitor hydroxyurea (HU) also induces ssDNA gaps [57]. HU inhibits ribonucleotide reductase, an enzyme responsible for making deoxynucleotides required for DNA synthesis, slowing down the replication fork, which triggers PrimPol repriming and generates extensive ssDNA gaps.

The biggest challenge with genotoxic therapies is the development of resistance. One of the reasons for resistance is suppression or repair of ssDNA gaps. For instance, BRCA-deficient cells rely on TLS for repairing post-replicative ssDNA gaps [28]. Therefore, up-regulation of post-replicative TLS, POLθ-mediated repair, or restored OF processing can suppress ssDNA gap accumulation and contribute to chemoresistance in BRCA-deficient cells [39]. Several combination strategies have been explored to overcome resistance by inhibiting ssDNA gap repair. For example, the TLS inhibitor JHRE06 is highly effective in killing BRCA-deficient cancer cells [68]. JHRE06 blocks TLS-mediated gap repair and thus synergizes with PARPi and cisplatin. Similarly, Polθ inhibitors like novobiocin or ART558 block alt-EJ and gap repair [39], while USP1 inhibitors destabilize replication forks, increasing gap formation and sensitizing cells to therapy [116].

The optimal combination of genotoxic drugs depends on the genetic makeup of the cancer cells and their mechanisms to therapy resistance. Sequencing can help identify mutations in the DNA repair genes like BRCA1/2. Transcriptomic profiling can indicate the up-regulation of DDT pathways that enable gap repair and contribute to resistance. Functional assays may be able to assess gap formation and fork stability. Combining these approaches can help stratify tumors and design personalized combination therapies. For example, combining PARPi or cisplatin with TLS inhibitors can be effective in tumors that rely on gap-filling mechanisms.

## Future directions

It is important to understand why some tumors are more sensitive to genotoxic chemotherapies than others in order to expand the benefit of gap-inducing drugs. Studies have shown that other genetic changes like PTEN mutations, IDH1/2 mutations, or overactive APOBEC3A can also contribute to an accumulation of ssDNA gaps even without BRCA deficiency [39]. By identifying pathways that suppress ssDNA gap accumulation, and which can be targeted to promote cell death, more patients could benefit from gap-inducing treatments. Additionally, it is not clear if the accumulation of gaps by itself can cause chemosensitivity or if DSB repair deficiency is ultimately required for tumor formation and treatment response. If ssDNA gaps alone underlie therapy response, this could mean that gap-inducing agents can be effective in tumors beyond BRCA deficiency. Future research should focus on accumulation of ssDNA gaps as a functional biomarker to understand tumor vulnerability. This will help identify targeted therapies and design combination treatments to prevent chemoresistance. Environmental factors can further complicate tumor vulnerability. Exposure to tobacco smoke, UV light, and reactive oxygen species has been shown to increase ssDNA gaps. Therefore, the future of genotoxic treatment lies in understanding ssDNA gap biology and how tumors exploit alternative pathways to survive.

## Data Availability

This is a review manuscript, no research is reported.

## Open Access

Open access for this article was enabled by the participation of The Pennsylvania State University in an all-inclusive Read & Publish agreement with Portland Press and the Biochemical Society under an agreement with the Big Ten Alliance.

## Abbreviations

APE1: AP endonuclease 1
BER: base excision repair
DDR: DNA damage response
DDT: DNA damage tolerance
DSBs: double-stranded DNA breaks
FEN1: Flap endonuclease 1
FP: fork protection
HR: homologous recombination
MRNIP: MRN-interacting protein
NER: nucleotide excision repair
OFs: Okazaki fragments
PARPi: PARP inhibitors
PCNA: proliferating cell nuclear antigen
PNKP: polynucleotide kinase phosphatase
PARG: poly(ADP-ribose) glycohydrolase
PARP1: poly(ADP-ribose) polymerase 1
RPA: replication protein A
SSB: single-stranded break
ssDNA gaps: single-stranded DNA gaps
TMEJ: theta-mediated end-joining
TIM: TIMELESS
TLS: translesion DNA synthesis
XRCC1: X-ray repair cross-complementing protein 1
